# Shotgun metagenomic sequencing reveals skin microbial variability from different facial sites

**DOI:** 10.3389/fmicb.2022.933189

**Published:** 2022-07-26

**Authors:** Qingzhen Wei, Zhiming Li, Zhenglong Gu, Xiao Liu, Jean Krutmann, Jiucun Wang, Jingjing Xia

**Affiliations:** ^1^Human Phenome Institute, School of Life Sciences, Fudan University, Shanghai, China; ^2^Ministry of Education Key Laboratory of Contemporary Anthropology, School of Life Sciences, Fudan University, Shanghai, China; ^3^BGI-Shenzhen, Shenzhen, China; ^4^Greater Bay Area Institute of Precision Medicine (Guangzhou), School of Life Sciences, Fudan University, Guangzhou, China; ^5^Shenzhen International Graduate School, Tsinghua University, Shenzhen, China; ^6^IUF-Leibniz Research Institute for Environmental Medicine, Düsseldorf, Germany; ^7^Department of Dermatology, Huashan Hospital, Fudan University, Shanghai, China; ^8^Research Unit of Dissecting the Population Genetics and Developing New Technologies for Treatment and Prevention of Skin Phenotypes and Dermatological Diseases (2019RU058), Chinese Academy of Medical Sciences, Beijing, China

**Keywords:** shotgun metagenomic sequencing, facial skin microbiome, Chinese, *Cutibacterium acnes* (*C. acnes*), *Propionibacterium acnes* bacteriophage, fine-scale, biogeography

## Abstract

Biogeography (body site) is known to be one of the main factors influencing the composition of the skin microbial community. However, site-associated microbial variability at a fine-scale level was not well-characterized since there was a lack of high-resolution recognition of facial microbiota across kingdoms by shotgun metagenomic sequencing. To investigate the explicit microbial variance in the human face, 822 shotgun metagenomic sequencing data from Han Chinese recently published by our group, in combination with 97 North American samples from NIH Human Microbiome Project (HMP), were reassessed. Metagenomic profiling of bacteria, fungi, and bacteriophages, as well as enriched function modules from three facial sites (forehead, cheek, and the back of the nose), was analyzed. The results revealed that skin microbial features were more alike in the forehead and cheek while varied from the back of the nose in terms of taxonomy and functionality. Analysis based on biogeographic theories suggested that neutral drift with niche selection from the host could possibly give rise to the variations. Of note, the abundance of porphyrin-producing species, i.e., *Cutibacterium acnes*, *Cutibacterium avidum*, *Cutibacterium granulosum*, and *Cutibacterium namnetense*, was all the highest in the back of the nose compared with the forehead/cheek, which was consistent with the highest porphyrin level on the nose in our population. Sequentially, the site-associated microbiome variance was confirmed in American populations; however, it was not entirely consistent. Furthermore, our data revealed correlation patterns between *Propionibacterium acnes* bacteriophages with genus *Cutibacterium* at different facial sites in both populations; however, *C. acnes* exhibited a distinct correlation with *P. acnes* bacteriophages in Americans/Chinese. Taken together, in this study, we explored the fine-scale facial site-associated changes in the skin microbiome and provided insight into the ecological processes underlying facial microbial variations.

## Introduction

The human skin is considered a complex ecosystem colonized with various microorganisms, including bacteria, fungi, and viruses, collectively termed “skin microbiota” (Grice and Segre, 2011). Balanced microbial community composition is essential for maintaining skin health (Byrd et al., 2018). However, this ecosystem turned out to be highly variable between individuals (Schommer and Gallo, 2013) and the factors responsible for the unique variability included endogenous host factors (host genetics, gender, and age) and exogenous environmental factors (lifestyle, hygiene routine, cosmetics, climate, and seasonality) (Grice and Segre, 2011; Boxberger et al., 2021).

Biogeography (body site) has been suggested as a major factor influencing the composition of the skin microbial community (Grice et al., 2009; Perez Perez et al., 2016; Wang et al., 2021). Characterization of spatiotemporal patterns in species distribution is a key task in biogeography and is also fundamental to explore the ecological and evolutionary processes shaping communities (Bahram et al., 2015). For skin microbiome, many studies favored to divide skin into four microenvironments (i.e., sebaceous, moist, dry, and foot) according to the physical and chemical properties of the anatomical sites (Oh et al., 2014). Although this classification was not delicate enough, some prominent features of microbial distribution pattern were well-characterized, for example, genus *Cutibacterium* and *Malassezia* favored oily (sebaceous) areas; genus *Staphylococcus* and *Corynebacterium* were predominant in moist areas while Gram-negative microorganisms favored dry areas (Grice et al., 2009; Chen and Tsao, 2013; Oh et al., 2014, 2016). However, microbial variance from anatomic sites at a more fine-scale level, for example, different sites from one’s face, was only partially understood (Lee et al., 2021). This is not trivial. Many facial conditions, exerting substantial adverse psychological and social influences, exhibited a clear and consistent site preference on the face, such as acne vulgaris and seborrheic dermatitis, prone to occur in oily areas with a rich supply of sebaceous glands (Williams et al., 2012; Tan and Bhate, 2015; Sparber et al., 2019), and rosacea often occurs in the central face such as the nose (Van Zuuren et al., 2011; Yigider et al., 2016). Therefore, it is valuable to learn about the microbial variance caused by this delicate body location, which may underlie the predisposition of skin dysbiosis conditions with site preference (Flowers and Grice, 2020).

Due to low microbial biomass from the skin (Chen et al., 2018), most studies deployed 16S rRNA sequencing and assessed only the bacterial community, leaving the fungal and viral communities largely unknown, particularly in the facial sites. To address this issue, we leveraged our shotgun metagenomic sequencing dataset generated from 822 Chinese samples (Li et al., 2021) and reassessed the data intensively, which allowed for more precise recognition of facial skin microbiota (forehead, cheek, and the back of the nose) across all kingdoms (bacteria, fungi, and viruses), in terms of microbial taxonomy and functionality. Sequentially, we reassessed 97 North-American metagenomic sequencing data from the Human Microbiome Project (HMP) (Oh et al., 2014) and compared the main features of the two populations. In particular, a series of *Propionibacterium acnes* bacteriophages, viral members which were considered important in regulating the balance of the microbiome, were assessed and highlighted.

## Materials and methods

### Study population

Ninety-seven North American samples from HMP (Oh et al., 2014) and 822 Han Chinese samples (Li et al., 2021) were selected. The datasets were downloaded from the integrated Human Skin Microbial Gene Catalog (iHSMGC). Detailed information about sampling, DNA preparation, and shotgun metagenomic sequencing can be obtained according to our previous study (Li et al., 2021).

### Statistical analysis

The Shannon index was used to represent the alpha diversity of the microbiome. Kruskal-Wallis test and Wilcoxon rank-sum test were used to assess the significance of the difference in three anatomical sites. Probability (*P*) values < 0.05 were considered to indicate statistically significant differences. *P*-values were adjusted using the false discovery rate (FDR) correction.

Beta diversity (principal coordinate analysis (PCoA) based on Bray-Curtis distances) was to characterize the microbial profile in different sites. The permutational multivariate analysis of variance (PERMANOVA) was used to assess the effect of different anatomical sites. We performed the analysis using the method implemented in the R package (vegan) and 1,000 times permutations to obtain the permuted *P*-value.

Linear discriminant analysis (LDA) effect size (LEfSe) was used to identify taxonomic differences between different anatomical sites. The threshold on the LDA score was set to 3.0.

Spearman correlation was carried out to investigate the existence of a correlation between *P. acnes* bacteriophages and four species that belong to the genus *Cutibacterium*, and the significance levels are *P < 0.05; ^**^P < 0.01; ^***^P < 0.001. *P*-values were adjusted using the FDR correction.

The LEfSe was completed using the Wekemo Bioincloud^1^. Another analysis was conducted using R (version 4.1.2).

Neutral community model analysis was used to explore ecological processes underlying microbial variations. Bray-Curtis distance of each site (FH forehead, CK cheek, NS nose) from the center was assessed using a classic model inferring genetic distance in molecular evolution (Li, 1997). Specifically, the distance to FH = [Distance (FH-NS) + Distance (FH-CK) – Distance (CK-NS)]/2; the distance to CK = [Distance (CK-NS) + Distance (FH-CK) – Distance (FH-NS)]/2; and the distance to NS = [Distance (FH-NS) + Distance (CK-NS) – Distance (FH-CK)]/2).


$$d_{F\,H}=\frac{D_{N\,S-F\,H}+D_{C\,K-F\,H}-D_{C\,K-N\,S}}{2}$$

$$d_{C\,K}=\frac{D_{C\,K-N\,S}+D_{F\,H-C\,K}-D_{F\,H-N\,S}}{2}$$

$$d_{N\,S}=\frac{D_{F\,H-N\,S}+D_{C\,K-N\,S}-D_{F\,H-C\,K}}{2}$$

## Results

### The back of the nose exhibited distinct microbial community composition from the forehead and cheek in the Chinese

We first investigated skin microbiome in three facial sites (forehead, cheek, and the back of the nose) from our population, in terms of the alpha diversity, microbial composition, and potential functionality.

**FIGURE 1:**
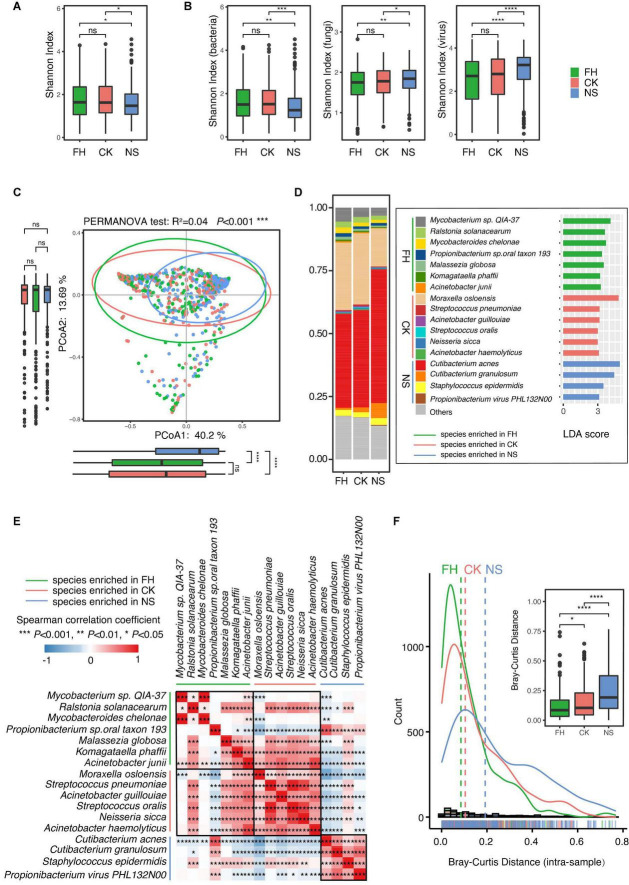
Comparative characterization of the skin microbiome present in three anatomical sites in Chinese samples. Boxplots comparing Shannon index of **(A)** overall skin microbiome and **(B)** bacteria, fungi, and virus microbiome of three anatomical sites in Chinese samples. **(C)** Principal coordinate analysis (PCoA) plot illustrating the comparison of the overall composition of skin microbiome between three sites in the Chinese population. The PERMANOVA test is used to determine significance. Boxplots indicate the distribution of samples along the PC1 and PC2. **(D)** Stack plot of the 17 differential species ranked by relative abundance. Linear discriminant analysis (LDA) effect size (LEfSe) histograms on the right showed the microbial comparisons of three anatomical sites, with an LDA threshold of 3.0. **(E)** Heat map of the Spearman’s correlation between differential species. **(F)** The distance distribution lines on the left showed the Bray-Curtis distance from the center of three facial sites to the forehead, to the cheek, and to the back of the nose, respectively. The boxplot on the right quantified and compared the difference. FH, the forehead; CK, the cheek; NS, the back of the nose. *P*-values were adjusted using the false discovery rate (FDR) correction. The significance levels are: ns, not significant, *P* > 0.05; *P < 0.05; ^**^P < 0.01; ^***^P < 0.001.

The overall alpha diversity, indicated by the Shannon index, was higher in the forehead and the cheek than in the nose, while the difference was not significant between the forehead and the cheek (Figure 1A). Furthermore, the Shannon index of each kingdom (bacteria, fungi, and viruses) from the three sites was also assessed. The results demonstrated that the back of the nose presented different microbial diversities from the other two sites, in regard to all kingdoms. However, in contrast to lower diversity in the bacterial community, the nose exhibited higher diversity in the fungal and viral community than that of the forehead and the cheek (Figure 1B). PCoA based on Bray-Curtis distance also confirmed a shift of nose microbiome from the other two sites, while the microbiome from the forehead and the cheek was more similar (PERMANOVA test, *R*^2^ = 0.04, *P* < 0.001) (Figure 1C).

To specify the differential species, LEfSe analysis was further carried out. The result revealed 17 site-associated dominant species across kingdoms in different facial sites: *Cutibacterium acnes*, *Cutibacterium granulosum*, *Staphylococcus epidermidis*, and *Propionibacterium* phage PHL132N00 were more abundant in the back of the nose; *Mycobacterium sp. QIA-37*, *Ralstonia solanacearum, Mycobacteroides chelonae, Propionibacterium sp. oral taxon 193, Malassezia globosa, Komagataella phaffii*, and *Acinetobacter junii* were more enriched in the forehead; and *Moraxella osloensis*, *Streptococcus pneumoniae*, *Acinetobacter guillouiae*, *Streptococcus oralis*, *Neisseria sicca*, and *Acinetobacter haemolyticus* were more abundant in the cheek (Figure 1D). The relative abundance of these 17 differential species varied significantly, especially between the nose and the other two sites (Figure 1D). Of note, the nose harbored clear higher amount of *C. acnes* and lower amount of *M. osloensis*. These two species were proven to be distinctive in nutrient demand: whereas *C. acnes* was high nutrient demanding and prone to the sebum-rich area, and *M. osloensis* was a non-fastidious bacterium that was able to grow in a mineral medium supplemented with a single organic carbon source (Juni, 1974, 2015). Correlation analysis further confirms this negative association between the two species. In addition, we found that a series of site-differential species were internally positive-correlated, whereas mostly negatively correlated with other site-prone species (Figure 1E). To further explore the possible mechanisms shaping the microbial biogeography, we conducted an analysis based on a neutral community model (Li, 1997), which is commonly applied to predict the assembly pattern of the communities and is favorable for the relative simplicity. By measuring the Bray-Curtis distance from the center of three sites, we found that the nose is much further from the center than the other two sites, whereas the distances for the other two are only marginally different (Figure 1F). A strict neutral drift would predict similar distances among all three lineages, indicating that selective forces (e.g., host selection) may exist in shaping the microbial variability, especially in the nose area.

### Shotgun metagenomic sequencing revealed that certain functionality underlies the site-associated microbiome variance in the Chinese population

**FIGURE 2:**
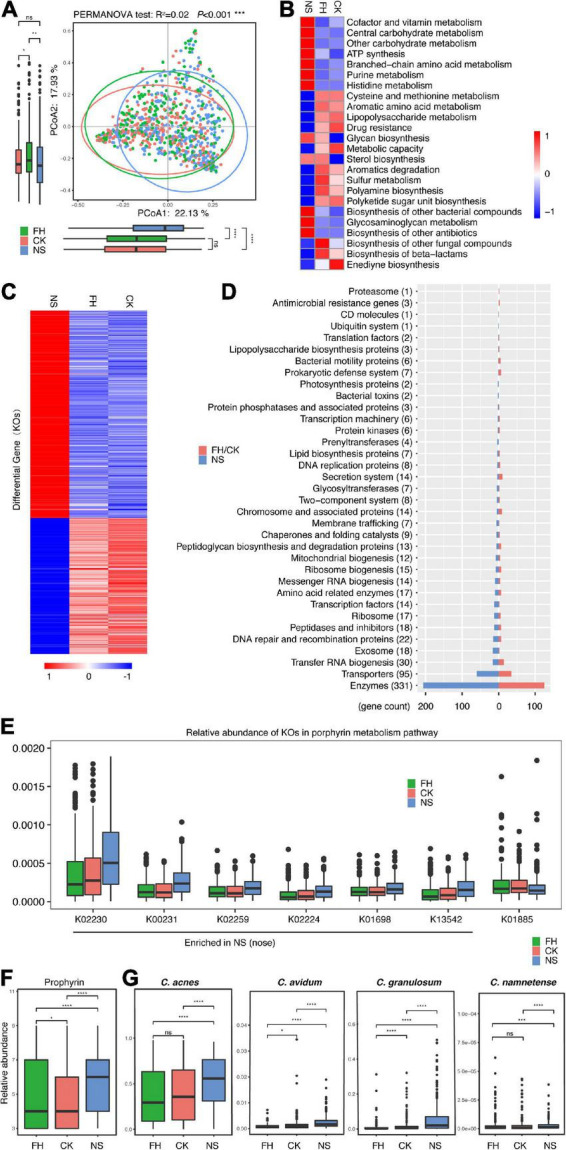
Comparative characterization of the skin microbiome present in three anatomical sites in American samples. **(A)** PCoA plot illustrating the comparison of the overall composition of skin microbiome between three sites in the Chinese population. The PERMANOVA test is used to determine significance. **(B)** Functional differences of the skin microbiome in three sites of Chinese samples. The heat map represents the relative abundance of function in three sites. **(C)** The heat map represents the relative abundance of 554 differential genes in three sites in Chinese samples. **(D)** Different categories that 554 differential genes belong to. **(E)** Boxplots comparing seven differential genes related to the porphyrin metabolism pathway in three anatomical sites (K02230: cobN, cobaltochelatase CobN; K00231: PPOX, protoporphyrinogen/coproporphyrinogen III oxidase; K02259: COX15, heme a synthase; K02224: cobB-cbiA; cobyrinic acid a,c-diamide synthase; K01698: hemB, porphobilinogen synthase; K13542: cobA-hemD, uroporphyrinogen III methyltransferase/synthase; K01885: EARS, glutamyl-tRNA synthetase). The relative abundance of facial porphyrin **(F)** and *Cutibacterium acnes*, *Cutibacterium avidum*, *Cutibacterium granulosum*, and *Cutibacterium namnetense* **(G)** in three sites. FH, the forehead; CK, the cheek; NS, the back of the nose. The significance levels are: ns, not significant, *P* > 0.05; *P < 0.05; ^**^P < 0.01; ^***^P < 0.001.

As shotgun metagenomic sequencing provided gene abundance information, we further assessed the functionality potentials of the microbiota located in these three anatomical sites. Overall, PCoA confirmed the variance in terms of gene features at the three sites (PERMANOVA test, *R*^2^ = 0.02, *P* < 0.001) (Figure 2A). The PC1 indicator of the PCoA showed a minor but significant difference between the back of the nose and the forehead/cheek. Furthermore, the heat map showed the relative abundance of 24 functional modules (Kyoto Encyclopedia of Genes and Genomes (KEGG) level C) enriched in the forehead and cheek while different from the back of the nose (Kruskal Wallis test, *P*-adjust < 0.05) (Figure 2B and Supplementary Table 1). Specifically, seven microbial functions of high gene abundance were all enriched in the nose, i.e., cofactor and vitamin metabolism, central carbohydrate metabolism, other carbohydrate metabolism, ATP synthesis, branched-chain amino acid metabolism, purine metabolism, and histidine metabolism. Other functions, many of which also related to metabolism, were more enriched in the forehead/cheek, such as serine and threonine metabolism, aromatic amino acid metabolism, lipopolysaccharide metabolism, and drug resistance.

More intensively, we identified 641 differential genes (out of 863 genes with relative abundance > 0.1%) (Kruskal Wallis test, *P*-adjust < 0.05). Notably, 554 of them (86.4%, Supplementary Table 2) showed a clear difference between the back of the nose and the forehead/cheek (Figure 2C), including 331 enzymes, 95 transporters, and other genes (Figure 2D and Supplementary Table 3). While 219 genes were more enriched in the forehead/cheek, 335 genes were more enriched in the nose. Interestingly, we found that there were seven differential genes, essential for the porphyrin metabolism, and six genes were more enriched in the back of the nose (Figure 2E). In fact, we observed that porphyrin levels, assessed with VISIA-CR pictures (Canfield Scientific Inc., Fairfield, NJ, USA), were the highest in the back of nose compared with the other two sites in our cohort (Figure 2F). Furthermore, it is known that several skin commensals were able to produce porphyrin, and while predominant from *C. acnes* (Shu et al., 2013; Spittaels et al., 2021), other *Propionibacterium* strains, such as *C. granulosum*, *Cutibacterium avidum*, and *Cutibacterium modestum* (previously, “*Propionibacterium humerusii*”) were also able to produce certain levels of porphyrin (Barnard et al., 2020). In consistent, our data revealed that the relative abundance of these porphyrin-producing species, i.e., *C. acnes*, *C. avidum*, *C. granulosum*, and *Cutibacterium namnetense*, were all the highest in the back of the nose compared with the forehead/cheek (Figure 2G).

### Facial site-associated microbiome variation is different between the Chinese and North American populations

**FIGURE 3:**
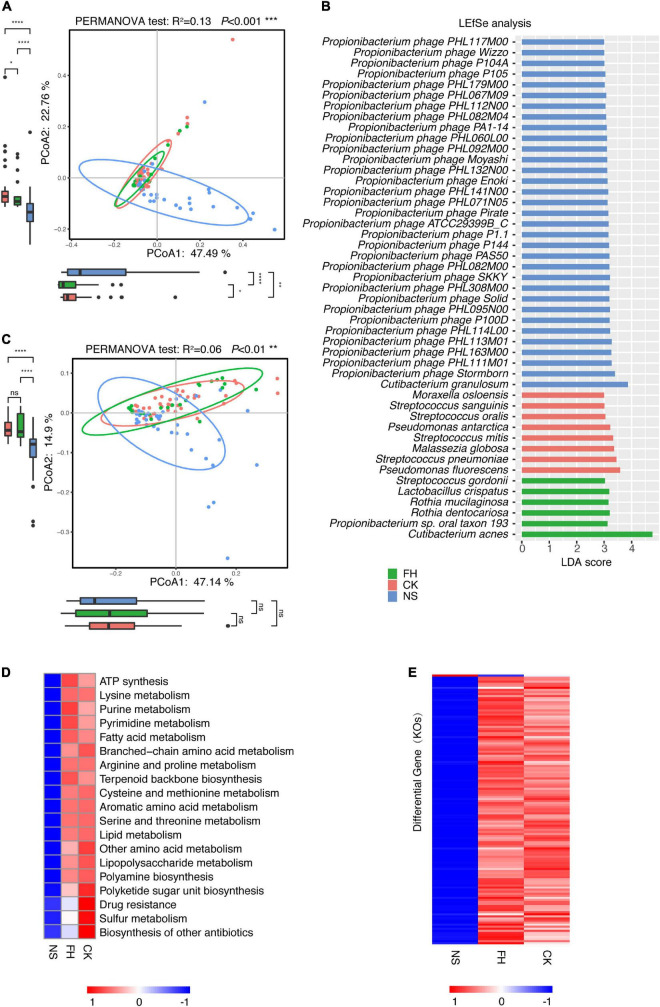
Comparative characterization of the skin microbiome present in three anatomical sites in American samples. **(A)** PCoA plot illustrating the comparison of the overall composition of skin microbiome between three sites in the American population. The PERMANOVA test is used to determine significance. Boxplots indicate the distribution of samples along the PC1 and PC2. **(B)** LEfSe histograms for the microbial comparisons of three anatomical sites, with an LDA threshold of 3.0. **(C)** PCoA plot illustrating the gene composition of skin microbiome between three sites in the American population. The PERMANOVA test is used to determine significance. **(D)** Functional differences of the skin microbiome in three sites of American samples. The heat map represents the relative abundance of function in three sites. **(E)** The heat map represents the relative abundance of 131 differential genes in three sites. FH, the forehead; CK, the cheek; NS, the back of the nose. *P*-values were adjusted using the FDR correction. The significance levels are: ns, not significant, *P* > 0.05; *P < 0.05; ^**^P < 0.01; ^***^P < 0.001.

Sequentially, we assessed the site-associated microbiome variance in the North American population. Overall, the PCoA suggested a microbiome variance existed among three facial sites (PERMANOVA test, *R*^2^ = 0.13, *P* < 0.001) and the back of the nose was different from the forehead/cheek (Figure 3A), consistent with the conclusion drawn from the Chinese population. Furthermore, LEfSe analysis revealed site-associated dominant species across the kingdoms.

In consistent, *M. osloensis*, *S. pneumoniae*, *S. oralis*, *Propionibacterium sp. oral taxon 193*, and *M. globosa* were more enriched in the forehead/cheek, whereas *C. granulosum* and a large series of *P. acnes* bacteriophages were enriched in the back of the nose in both populations (Figure 3B). Of note, *C. acnes* was more enriched in the forehead in Americans, which contrasted with the highest abundance in the back of the nose in Chinese. *S. epidermidis* showed more enrichment in the back of the nose in Chinese, but no site difference in Americans.

Based on the gene abundance, the PCoA also showed that the forehead and the cheek were much more similar but both different from the back of the nose (PERMANOVA test, *R*^2^ = 0.06, *P* < 0.001) (Figure 3C). Of note, 19 microbial functions (KEGG level C) were found to be significantly different in three sites (Kruskal Wallis test, *P*-adjust < 0.05) (Figure 3D and Supplementary Table 4). The heat map showed the relative abundance of differential functions from the three facial sites. Specifically, several microbial functions, such as cysteine and methionine metabolism, aromatic amino acid metabolism, lipopolysaccharide metabolism, drug resistance, sulfur metabolism, polyamine biosynthesis, and polyketide sugar unit biosynthesis were also higher in the forehead/cheek in the Chinese samples.

In Americans, there were 145 site-associated differential genes (Kruskal Wallis test, *P*-adjust < 0.05), and 131 of them (90.3%) showed similar abundance between the forehead and cheek but significantly different from the back of the nose. Among these 131 genes, only K17316 (glucose/mannose transport system permease protein) was more enriched in the back of the nose, while the rests were more enriched in the forehead/cheek (Figure 3E and Supplementary Table 5).

### A distinct correlation between *Propionibacterium acnes* bacteriophages and *Cutibacterium acnes* was observed in the two populations

*Propionibacterium acnes* bacteriophages, members of the viral community, are dominant bacteriophages that existed in the skin microbiota, especially in the pilosebaceous unit (Liu et al., 2015). These bacteriophages were able to play an important role in maintaining the balance of the microbial community (Liu et al., 2015). However, the association between these bacteriophages with other skin microbiota was rarely studied.

**FIGURE 4:**
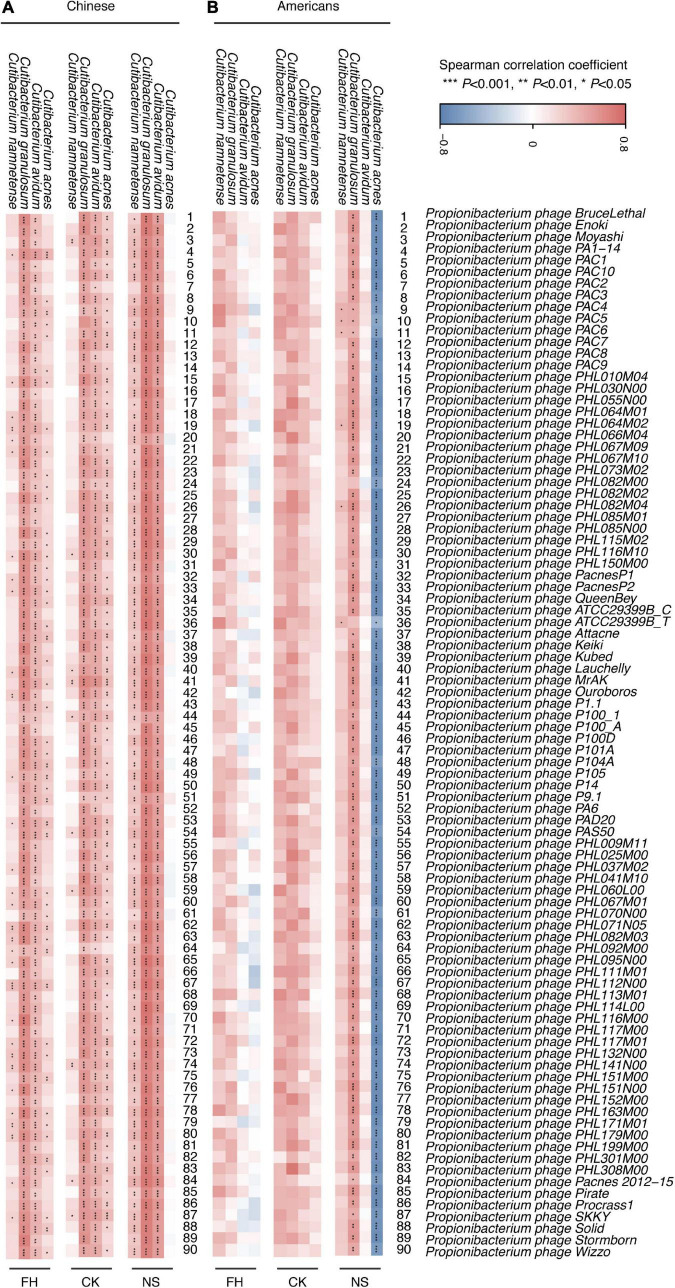
The correlation between *Cutibacterium* and *Propionibacterium acnes* bacteriophages in Chinese and American samples. Heat map of the results of the Spearman correlation between four species in genus *Cutibacterium* and *P. acnes* bacteriophages in three sites and in Chinese **(A)** and American **(B)** samples. FH, the forehead; CK, the cheek; NS, the back of the nose. *P*-values were adjusted using the FDR correction. The significance levels in the Spearman correlation are: *P < 0.05; ^**^P < 0.01; ^****^P < 0.001.

In this study, we assessed the correlation between *P. acnes* bacteriophages and all detectable species from the genus *Cutibacterium* (Dekio et al., 2021), in three sites of two populations. In general, the Chinese showed more correlations in three sites compared with the Americans (Figure 4). For Chinese populations, the forehead and the cheek presented mostly consistent positive correlation between genus *Cutibacterium*, particular *C. acnes*, *C. granulosum*, and *C. avidum* with most detected *P. acnes* bacteriophages; while in the back of the nose, *C. acnes* and *P. acnes* bacteriophages showed no significant correlation, but the correlations between *C. namnetense* and bacteriophages were significant (Figure 4A). In contrast, most correlations in the forehead/cheek showed similar trends but not significant in Americans. In particular, *P. acnes* bacteriophages exhibited a consistent positive correlation with *C. granulosum*, but a significant negative correlation with *C. acnes* in the back of the nose in Americans (Figure 4B).

## Discussion

In this study, we centered on addressing site-associated microbiome variance in Chinese facial skin, by intensively reassessing our shotgun metagenomic dataset generated from 822 Chinese samples (Li et al., 2021). Overall, our data revealed that microbial features in the back of the nose were distinctive from the forehead and cheek in Chinese. Furthermore, we confirmed a similar site-associated microbial pattern in the North American population, although varied in detail.

It is long known that biogeography (body site) is a major factor influencing the composition of the skin microbial community (Grice et al., 2009; Perez Perez et al., 2016; Wang et al., 2021). However, there was very limited understanding of the mechanisms shaping microbial biogeography as it is often rather difficult to determine the relative importance of drift, dispersal, speciation, and selection, the four processes (mechanisms) determining the patterns of biogeography and community dynamics (Ma, 2021). Nevertheless, there were several studies worked on the relative significance of stochastic neutral forces and deterministic niche selection and brought us new insights into the mechanisms, shaping the biogeography of the human microbiome (Ma et al., 2018; Tong et al., 2019; Ma, 2021). For example, an analysis of a multi-site microbiome, covering five major habitats (i.e., airway, oral, gut, skin, and urogenital) suggested the relative significance of stochastic neutral forces and deterministic niche selection in shaping the biogeography of the human microbiome (Ma et al., 2018). Another study also suggested that while skin mycobiome assembly is a predominantly neutral process, taxa that could be under the influence of selective forces (e.g., host selection) are potentially key to the structure of a community network (Tong et al., 2019). In this study, we observed a similar pattern that fit to a “nutrient-drive” model by explaining the site-associated microbial disparity.

In contrast, addressing site-associated microbial variation at a fine-scale level is important for digging the niche selection pressure for the skin microbiome. Multiple variables, such as hygiene routine, cosmetics, climate, and seasonality, which were known to impact microbial niche conditions (Grice and Segre, 2011; Harris-Tryon and Grice, 2022), were well-controlled in this adjacent subsite area and thereby substantially facilitate decoding the microbial variation. Learned from classical ecology, the selection pressures for the ecosystem include resource availability (presence of nutrients), environmental conditions (temperature, geographical access), and biological factors (predators and pathogens) (Williams, 1996). In this study, we revealed a series of site-prone species, many of which were previously proven to be distinct in nutrient requirements, i.e., *C. acnes as* high nutrient-demand, and *M. osloensis* as low nutrient-demand species able to grow in a mineral medium supplemented with a single organic carbon source (Juni, 1974; Juni and Bøvre, 2015). Furthermore, we revealed that site-associated species correlated with each in pattern, suggesting that specific interactions between species underlie the formation of networks to compete in the niche occupation. In turn, the colonization of microbiota in different sites may also reflect niche conditions. In consistent, *C. acnes* tends to colonize in oily areas, and the abundance increases with the sebum level (Mukherjee et al., 2016). A study in Korean women revealed higher sebum secretion in the nose than in the forehead and cheek (Youn et al., 2005), consistent with the higher abundance of *C. acnes* in the nose than in the forehead/cheek in our study. However, the relative abundance of *C. acnes* was demonstrated the highest in the forehead in Americans, which may be due to the ethnical differences in regard to delicate anatomic structures, such as the count and size of sebaceous glands and physiological phenotypes (Rawlings, 2006; Voegeli et al., 2019).

In addition, our data revealed site-associated microbial features not only in taxonomical composition but also in functionality. In Chinese, the carbohydrate metabolism of microbiota was more enriched in the back of the nose, which is consistent with the fact that *C. acnes* utilized carbohydrates as the main carbon source (Li et al., 2021). In contrast, *M. osloensis* was incapable of utilizing any carbohydrates or possessing any saccharolytic activity but strictly depend on other carbon sources such as acetic or lactic acid (Baumann et al., 1968; Juni, 1974; Moss et al., 1988; Juni and Bøvre, 2015).

In our study, *M. osloensis* was the most abundant differential species in the cheek, which exhibited the lowest hydration level (Lee et al., 2013; Machkova et al., 2018) as well as the sebum level (Youn et al., 2005). In both populations, some function modules were more enriched in the forehead/cheek compared with the back of the nose, including sulfur metabolism, cysteine and methionine metabolism, aromatic amino acid metabolism, polyketide sugar unit biosynthesis, and drug resistance, which may imply a more challenged or competitive environment for microbes to adapt in the forehead/cheek than the nose. For example, sulfur is an essential nutrient and can be metabolized into the sulfur-containing amino acids (cysteine and methionine) in microorganisms, protecting against oxidative and environmental stresses such as dryness (Ernst, 1998; Yi et al., 2010; Chan et al., 2019).

In this study, some microbial composition-associated skin feature was validated, i.e., the enrichment of genes in porphyrin metabolism in the nose was demonstrated to link to the abundance of porphyrin-producing species, which were further proven to be positively associated with high porphyrin level on the nose. It is known that bacterial porphyrins are considered to be pro-inflammatory and linked to inflammatory skin diseases (Schaller et al., 2005). Our findings may underlie this site preference for specific inflammatory skin conditions, such as acne vulgaris or rosacea.

Furthermore, one of the highlights of this study was that we were able to explore the composition of other communities, in addition to bacteria, in these facial sites. Bacteriophages, viruses that infect corresponding host bacteria, may play an important regulatory role in human skin health (Liu et al., 2015). However, the interaction between bacteriophage with other skin microbiota is rarely known. In this study, we found that *C. granulosum* and various *P. acnes* bacteriophages were enriched in the nose in both populations. Furthermore, there was an intriguing correlation pattern between *P. acnes* bacteriophages with genus *Cutibacterium* at different facial sites in both populations. Of note, *C. acnes* demonstrated a distinct correlation with *P. acnes* bacteriophages in American/Chinese. It is known that the distribution of *P. acnes* bacteriophages depends on their specific host species (Jonczyk-Matysiak et al., 2017) and recent studies revealed the complexity of different lineages of *C. acnes* on the skin (Dekio et al., 2021; Conwill et al., 2022). These all implied that the significance of more deep sequencing was needed in the future to address complicated correlations.

## Data availability statement

The original contributions presented in this study are included in the article/supplementary material, further inquiries can be directed to the corresponding authors.

## Ethics statement

The studies involving human participants were reviewed and approved by the Ethics Committee, School of Life Sciences, Fudan University, China. The patients/participants provided their written informed consent to participate in this study.

## Author contributions

QZW and ZML: formal analysis and visualization. QZW and JJX: writing – original draft preparation. QZW, JJX, ZLG, and JK: writing – review and editing. JJX, JCW, ZLG, JK, and XL: scientific supervision. JJX and JCW: funding acquisition. All authors have read and agreed to the published version of the manuscript.

## Footnotes

1
https://www.bioincloud.tech

## References

[B1] Bahram M. Peay K. G. Tedersoo L. (2015). Local-scale biogeography and spatiotemporal variability in communities of mycorrhizal fungi. *New Phytol.* 205 1454–1463. 10.1111/nph.13206 25767850

[B2] Barnard E. Johnson T. Ngo T. Arora U. Leuterio G. Mcdowell A. (2020). Porphyrin Production and Regulation in Cutaneous Propionibacteria. *MSphere* 2020:5. 10.1128/mSphere.00793-19 31941813 PMC6968654

[B3] Baumann P. Doudoroff M. Stanier R. Y. (1968). Study of the *Moraxella* group. I. Genus *Moraxella* and the Neisseria catarrhalis group. *J. Bacteriol.* 95 58–73. 10.1128/jb.95.1.58-73.1968 4866103 PMC251972

[B4] Boxberger M. Cenizo V. Cassir N. La Scola B. (2021). Challenges in exploring and manipulating the human skin microbiome. *Microbiome* 9:125. 10.1186/s40168-021-01062-5 34053468 PMC8166136

[B5] Byrd A. L. Belkaid Y. Segre J. A. (2018). The human skin microbiome. *Nat. Rev. Microbiol.* 16 143–155. 10.1038/nrmicro.2017.157 29332945

[B6] Chan K. X. Phua S. Y. Van Breusegem F. (2019). Secondary sulfur metabolism in cellular signalling and oxidative stress responses. *J. Exp. Bot.* 70 4237–4250. 10.1093/jxb/erz119 30868163

[B7] Chen Y. E. Fischbach M. A. Belkaid Y. (2018). Skin microbiota-host interactions. *Nature* 553 427–436. 10.1038/nature25177 29364286 PMC6075667

[B8] Chen Y. E. Tsao H. (2013). The skin microbiome: current perspectives and future challenges. *J. Am. Acad. Dermatol.* 69 143–155. 23489584 10.1016/j.jaad.2013.01.016 PMC3686918

[B9] Conwill A. Kuan A. C. Damerla R. Poret A. J. Baker J. S. Tripp A. D. (2022). Anatomy promotes neutral coexistence of strains in the human skin microbiome. *Cell Host Microbe* 30 171–182e177. 10.1016/j.chom.2021.12.007 34995483 PMC8831475

[B10] Dekio I. Asahina A. Shah H. N. (2021). Unravelling the eco-specificity and pathophysiological properties of *Cutibacterium* species in the light of recent taxonomic changes. *Anaerobe* 71:102411. 10.1016/j.anaerobe.2021.102411 34265438

[B11] Ernst W. H. (1998). Sulfur metabolism in higher plants: potential for phytoremediation. *Biodegradation* 9 311–318. 10.1023/A:1008250827209 10022074

[B12] Flowers L. Grice E. A. (2020). The Skin Microbiota: Balancing Risk and Reward. *Cell Host Microbe* 28 190–200. 10.1016/j.chom.2020.06.017 32791112 PMC7444652

[B13] Grice E. A. Kong H. H. Conlan S. Deming C. B. Davis J. Young A. C. (2009). Topographical and temporal diversity of the human skin microbiome. *Science* 324 1190–1192. 10.1126/science.1171700 19478181 PMC2805064

[B14] Grice E. A. Segre J. A. (2011). The skin microbiome. *Nat. Rev. Microbiol.* 9 244–253. 10.1038/nrmicro2537 21407241 PMC3535073

[B15] Harris-Tryon T. A. Grice E. A. (2022). Microbiota and maintenance of skin barrier function. *Science* 376 940–945. 10.1126/science.abo0693 35617415

[B16] Jonczyk-Matysiak E. Weber-Dabrowska B. Zaczek M. Miedzybrodzki R. Letkiewicz S. Lusiak-Szelchowska M. (2017). Prospects of Phage Application in the Treatment of Acne Caused by *Propionibacterium acnes*. *Front. Microbiol.* 8:164. 10.3389/fmicb.2017.00164 28228751 PMC5296327

[B17] Juni E. (1974). Simple genetic transformation assay for rapid diagnosis of *Moraxella osloensis*. *Appl. Microbiol.* 27 16–24. 10.1128/am.27.1.16-24.1974 4589126 PMC379961

[B18] Juni E. Bøvre K. (2015). “Moraxella,” in *Bergey’s Manual of Systematics of Archaea and Bacteria*, (Hoboken, NJ: Wiley), 1–17. 10.1002/9781118960608.gbm01204

[B19] Juni E. B. K. (2015). *Bergey’s Manual of Systematics of Archaea and Bacteria.* Hoboken, NJ: John Wiley & Sons, Inc.

[B20] Lee H. Jeong J. Oh Y. Lee C. J. Mun S. Lee D. G. (2021). Comparative analysis of human facial skin microbiome between topical sites compared to entire face. *Genes Gen.* 43 1483–1495. 10.1007/s13258-021-01180-2 34734352

[B21] Lee M. R. Nam G. W. Jung Y. C. Park S. Y. Han J. Y. Cho J. C. (2013). Comparison of the skin biophysical parameters of Southeast Asia females: forehead-cheek and ethnic groups. *J. Eur. Acad. Dermatol. Venereol.* 27 1521–1526. 10.1111/jdv.12042 23216687

[B22] Li W.-H. (1997). *Molecular Evolution.* Sunderland, MA: Sinauer Associates.

[B23] Li Z. Xia J. Jiang L. Tan Y. An Y. Zhu X. (2021). Characterization of the human skin resistome and identification of two microbiota cutotypes. *Microbiome* 9:47. 10.1186/s40168-020-00995-7 33597039 PMC7890624

[B24] Liu J. Yan R. Zhong Q. Ngo S. Bangayan N. J. Nguyen L. (2015). The diversity and host interactions of *propionibacterium acnes* bacteriophages on human skin. *ISME J* 9 2078–2093. 10.1038/ismej.2015.47 25848871 PMC4542041

[B25] Ma Z. Li L. Li W. (2018). Assessing and interpreting the within-body biogeography of human microbiome diversity. *Front. Microbiol.* 9:1619. 10.3389/fmicb.2018.01619 30131772 PMC6090070

[B26] Ma Z. S. (2021). Niche-neutral theoretic approach to mechanisms underlying the biodiversity and biogeography of human microbiomes. *Evol. Appl.* 14 322–334. 10.1111/eva.13116 33664779 PMC7896709

[B27] Machkova L. Svadlak D. Doleckova I. (2018). A comprehensive *in vivo* study of Caucasian facial skin parameters on 442 women. *Arch. Dermatol. Res.* 310 691–699. 10.1007/s00403-018-1860-6 30167813

[B28] Moss C. W. Wallace P. L. Hollis D. G. Weaver R. E. (1988). Cultural and chemical characterization of CDC groups EO-2, M-5, and M-6, *Moraxella* (*Moraxella*) species, Oligella urethralis, Acinetobacter species, and Psychrobacter immobilis. *J. Clin. Microbiol.* 26 484–492. 10.1128/jcm.26.3.484-492.1988 3356788 PMC266318

[B29] Mukherjee S. Mitra R. Maitra A. Gupta S. Kumaran S. Chakrabortty A. (2016). Sebum and hydration levels in specific regions of human face significantly predict the nature and diversity of facial skin microbiome. *Sci. Rep.* 6:36062. 10.1038/srep36062 27786295 PMC5081537

[B30] Oh J. Byrd A. L. Deming C. Conlan S. Kong H. H. Segre J. A. (2014). Biogeography and individuality shape function in the human skin metagenome. *Nature* 514 59–64. 10.1038/nature13786 25279917 PMC4185404

[B31] Oh J. Byrd A. L. Park M. Program N. C. S. Kong H. H. Segre J. A. (2016). Temporal stability of the human skin microbiome. *Cell* 165 854–866. 27153496 10.1016/j.cell.2016.04.008 PMC4860256

[B32] Perez Perez G. I. Gao Z. Jourdain R. Ramirez J. Gany F. Clavaud C. (2016). Body site is a more determinant factor than human population diversity in the healthy skin microbiome. *PLoS One* 11:e0151990. 10.1371/journal.pone.0151990 27088867 PMC4835103

[B33] Rawlings A. V. (2006). Ethnic skin types: are there differences in skin structure and function? *Int. J. Cosmet. Sci.* 28 79–93. 10.1111/j.1467-2494.2006.00302.x 18492142

[B34] Schaller M. Loewenstein M. Borelli C. Jacob K. Vogeser M. Burgdorf W. H. C. (2005). Induction of a chemoattractive proinflammatory cytokine response after stimulation of keratinocytes with *propionibacterium acnes* and coproporphyrin III. *Br. J. Dermat.* 153 66–71. 10.1111/j.1365-2133.2005.06530.x 16029328

[B35] Schommer N. N. Gallo R. L. (2013). Structure and function of the human skin microbiome. *Trends Microbiol.* 21 660–668. 10.1016/j.tim.2013.10.001 24238601 PMC4744460

[B36] Shu M. Kuo S. Wang Y. Jiang Y. Liu Y. T. Gallo R. L. (2013). Porphyrin metabolisms in human skin commensal *propionibacterium acnes* bacteria: potential application to monitor human radiation risk. *Curr. Med. Chem.* 20 562–568. 10.2174/0929867311320040007 23231351 PMC3878905

[B37] Sparber F. De Gregorio C. Steckholzer S. Ferreira F. M. Dolowschiak T. Ruchti F. (2019). The skin commensal yeast malassezia triggers a type 17 response that coordinates anti-fungal immunity and exacerbates skin inflammation. *Cell Host Microbe* 2019:25. 10.1016/j.chom.2019.02.002 30870621

[B38] Spittaels K. J. Van Uytfanghe K. Zouboulis C. C. Stove C. Crabbe A. Coenye T. (2021). Porphyrins produced by acneic *cutibacterium acnes* strains activate the inflammasome by inducing K(+) leakage. *iScience* 24:102575. 10.1016/j.isci.2021.102575 34151228 PMC8188554

[B39] Tan J. K. L. Bhate K. (2015). A global perspective on the epidemiology of acne. *Br. J. Dermat.* 172(Suppl.):1. 10.1111/bjd.13462 25597339

[B40] Tong X. Leung M. H. Y. Wilkins D. Cheung H. H. L. Lee P. K. H. (2019). Neutral processes drive seasonal assembly of the skin mycobiome. *mSystems* 2019:4. 10.1128/mSystems.00004-19 30944878 PMC6435813

[B41] Van Zuuren E. J. Kramer S. Carter B. Graber M. A. Fedorowicz Z. (2011). Interventions for rosacea. *Cochrane Database Syst. Rev.* 2011:CD003262. 10.1002/14651858.CD003262.pub4 21412882

[B42] Voegeli R. Gierschendorf J. Summers B. Rawlings A. V. (2019). Facial skin mapping: from single point bio-instrumental evaluation to continuous visualization of skin hydration, barrier function, skin surface pH, and sebum in different ethnic skin types. *Int. J. Cosmet. Sci.* 41 411–424. 10.1111/ics.12562 31325176 PMC6851972

[B43] Wang Y. Yu Q. Zhou R. Feng T. Hilal M. G. Li H. (2021). Nationality and body location alter human skin microbiome. *Appl. Microb. Biotechnol.* 105 5241–5256. 10.1007/s00253-021-11387-8 34125277

[B44] Williams G. C. (1996). *Natural Selection, Ecology, and Morphogenesis in Adaptation and Natural Selection.* Princeton: Princeton University Press, 56–91.

[B45] Williams H. C. Dellavalle R. P. Garner S. (2012). Acne vulgaris. *Lancet* 379 361–372. 10.1016/S0140-6736(11)60321-8 21880356

[B46] Yi H. Ravilious G. E. Galant A. Krishnan H. B. Jez J. M. (2010). From sulfur to homoglutathione: thiol metabolism in soybean. *Amino Acids* 39 963–978. 10.1007/s00726-010-0572-9 20364282

[B47] Yigider A. P. Kayhan F. T. Yigit O. Kavak A. Cingi C. (2016). Skin diseases of the nose. *Am. J. Rhinol. Allergy* 30 83–90. 10.2500/ajra.2016.30.4318 27216341

[B48] Youn S. W. Na J. I. Choi S. Y. Huh C. H. Park K. C. (2005). Regional and seasonal variations in facial sebum secretions: a proposal for the definition of combination skin type. *Skin Res.* T*echnol.* 11 189–195. 10.1111/j.1600-0846.2005.00119.x 15998330

